# Structure and Characterization of a Covalent Inhibitor of Src Kinase

**DOI:** 10.3389/fmolb.2020.00081

**Published:** 2020-05-19

**Authors:** Deepak Gurbani, Guangyan Du, Nathaniel J. Henning, Suman Rao, Asim K. Bera, Tinghu Zhang, Nathanael S. Gray, Kenneth D. Westover

**Affiliations:** ^1^Departments of Biochemistry and Radiation Oncology, The University of Texas Southwestern Medical Center at Dallas, Dallas, TX, United States; ^2^Department of Biological Chemistry and Molecular Pharmacology, Harvard Medical School, Boston, MA, United States; ^3^Department of Cancer Biology, Dana Farber Cancer Institute, Boston, MA, United States; ^4^Harvard Program in Therapeutic Science (HiTS), Harvard Medical School, Boston, MA, United States

**Keywords:** src kinase, cancer, dasatinib, selectivity, irreversible inhibitor

## Abstract

Unregulated Src activity promotes malignant processes in cancer, but no Src-directed targeted therapies are used clinically, possibly because early Src inhibitors produce off-target effects leading to toxicity. Improved selective Src inhibitors may enable Src-directed therapies. Previously, we reported an irreversible Src inhibitor, DGY-06-116, based on the hybridization of dasatinib and a promiscuous covalent kinase probe SM1-71. Here, we report biochemical and biophysical characterization of this compound. An x-ray co-crystal structure of DGY-06-116: Src shows a covalent interaction with the kinase p-loop and occupancy of the back hydrophobic kinase pocket, explaining its high potency, and selectivity. However, a reversible analog also shows similar potency. Kinetic analysis shows a slow inactivation rate compared to other clinically approved covalent kinase inhibitors, consistent with a need for p-loop movement prior to covalent bond formation. Overall, these results suggest that a strong reversible interaction is required to allow sufficient time for the covalent reaction to occur. Further optimization of the covalent linker may improve the kinetics of covalent bond formation.

## Introduction

*SRC* was among the first oncogenes to be discovered (Stehelin et al., 1976) and encodes a non-receptor protein tyrosine kinase that regulates many cancer-related cellular processes including mitogenesis, angiogenesis, adhesion, invasion, migration, and survival (Sen and Johnson, 2011). Src activity drives malignant phenotypes in hematologic and solid cancers including breast, prostate, lung, colorectal, and pancreatic cancer (Araujo and Logothetis, 2010; de Felice et al., 2016; Appel et al., 2017). Genetic ablation of Src in animal models reverses cancer phenotypes without systemic toxicity (Trevino et al., 2006; Ammer et al., 2009; Marcotte et al., 2012), suggesting that Src inhibition may be effective in treating certain cancers (Araujo and Logothetis, 2009; Zhang et al., 2009; Chen et al., 2014; Anderson et al., 2017; Appel et al., 2017). Src has also been implicated in cancer drug resistance (Carretero et al., 2010; Sen et al., 2011). Nevertheless, selective Src inhibition has not been demonstrated as a driver of efficacy for any of the clinically used multi-targeted Src drugs.

Dasatinib and bosutinib inhibit multiple kinases including Src, but are approved as anti-Bcr-Abl therapies to treat chronic myelogenous leukemia and acute lymphoblastic leukemia (Shah et al., 2010; Keskin et al., 2016; Cortes et al., 2019). Src-directed trials using dasatinib failed in part due to dose-limiting toxicity (Araujo and Logothetis, 2010; Algazi et al., 2012; Araujo et al., 2012; Secord et al., 2012; Sharma et al., 2012; Schott et al., 2016) including grade 3 to 4 diarrhea, thrombocytopenia, neutropenia, and anemia (Buglio et al., 2012; Daud et al., 2012). These toxicities may be due to the multi-targeted nature of these compounds that also inhibit members of the Src family of kinases (SFKs), Bcr-Abl, c-Kit, PDGFR, c-Fms, and EphA2. Improved Src inhibitors with better selectivity may enable Src-directed cancer therapies.

**Figure 1:**
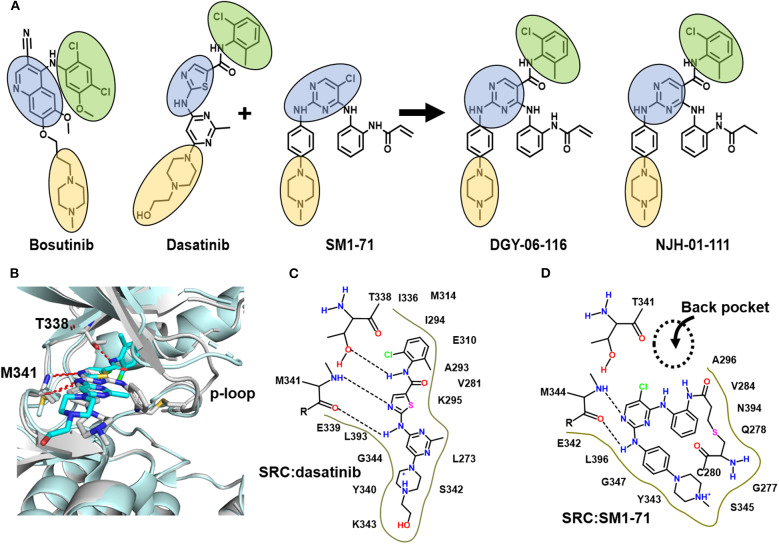
DGY-06-116 is a hybrid of dasatinib and SM171. **(A)** Src inhibitors are composed of a kinase hinge-binding component (blue), back pocket-binding component (green), and solvent-exposed component (yellow). DGY-06-116 (second from right) resulted from hybridizing the SM1-71 core (third from left) with the back-pocket component of dasatinib (second from left). NJH-01-111 (first from right) is a non-covalent analog. **(B)** Superposition of Src-dasatinib (PDB: 3G5D; cyan) and Src-SM1-71 (PDB: 6ATE; gray) structures suggested that the substitution would be tolerated. Schematic representation of interactions between Src and **(C)** dasatinib or **(D)** SM1-71. Dotted lines are hydrogen bonds.

Engineering selectivity into Src inhibitors is challenging because of the high degree of sequence homology between Src family members and other receptor tyrosine kinases (Duan et al., 2014; Elias and Ditzel, 2015). One strategy for achieving selectivity in kinases is to utilize covalent chemistry, targeting non-conserved cysteines near the inhibitor binding site. Prior work showed that Src, in particular, is amenable to this approach by targeting non-conserved cysteines in the p-loop (Kwarcinski et al., 2012). In that work, promiscuous scaffolds, including the dasatinib scaffold, were derivatized to include reactive warheads that could react with p-loop cysteines, resulting in enhanced selectivity for kinases that include a p-loop cysteine. The work generated hypotheses regarding the importance of p-loop dynamics for this class of inhibitor, but structural data were not reported. Recently, we found an opportunity to build upon this strategy when we found that SM1-71, a 2, 4-disubstituted pyrimidine that includes a cysteine-reactive warhead, can covalently modify 23 different kinases including Src. Our Src-SM1-71 crystal structure (PDB: 6ATE) revealed the Src p-loop in a kinked conformation (Rao et al., 2019). We subsequently showed that SM-1-71 could be optimized for Src inhibition by hybridization with dasatinib (Figures 1A–D, Figure S1; Du et al., 2020). Here, we present formal biochemical, biophysical, and structural characterization of Src inhibition by DGY-06-116.

## Results

### DGY-06-116 Potently Inhibits Src Kinase Activity

**Figure 2:**
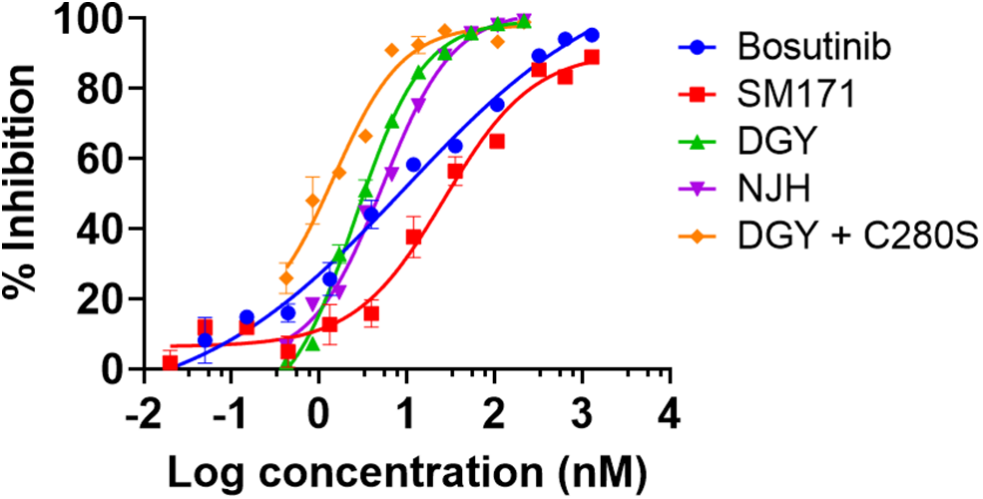
DGY-06-116 inhibits Src enzymatic activity. DGY-06-116 potency at 1 h exceeds SM1-71 and bosutinib. However, a non-covalent analog NJH-01-111 also shows potent activity. DGY-06-116 also potently inhibits Src^C280S^. Error bars are the standard deviation of three replicates.

DGY-06-116 was previously characterized for the ability to bind Src (Du et al., 2020). Here, we evaluated the relative potency of inhibitors on Src enzymatic activity using a mobility shift assay (MSA), which measures phosphorylation of a peptide substrate of Src. Given that covalent inhibitors can show time-dependent effects on IC_50_ values, we use a 1-h time point for all samples so relative potencies are comparable. At 1-h incubation, DGY-06-116 showed an IC_50_, 1 h of 2.6 nM. This was substantially better than SM1-71 (IC_50_, 1 h of 26.6 nM), bosutinib (IC_50_ of 9.5 nM), and its non-covalent analog NJH-01-111 (IC_50_ of 5.3 nM) (Figure 2). To estimate the contribution of covalent binding to the overall potency of DGY-06-116, we also tested Src^C280S^, which cannot form a covalent bond because of the cysteine-to-serine mutation. Src^C280S^ showed excellent kinase activity, although specific activity was less (~50%) than that of wild-type protein (Figure S2B). DGY-06-116 showed comparable IC_50_, 1 h values for mutant and wild-type Src (Figure 2), suggesting that reversible binding substantially contributes to the potency of this compound. To confirm this, we also tested a non-covalent analog of DGY-06-116, NJH-01-111, in which the acrylamide warhead is replaced with propionamide (Figure 1A). The IC_50_ of NJH-01-111 could not be distinguished from DGY-06-116, confirming that the core scaffold supports the potency of this compound class (Figure 2).

### Structure of the Src-DGY-06-116 Complex

**Figure 3:**
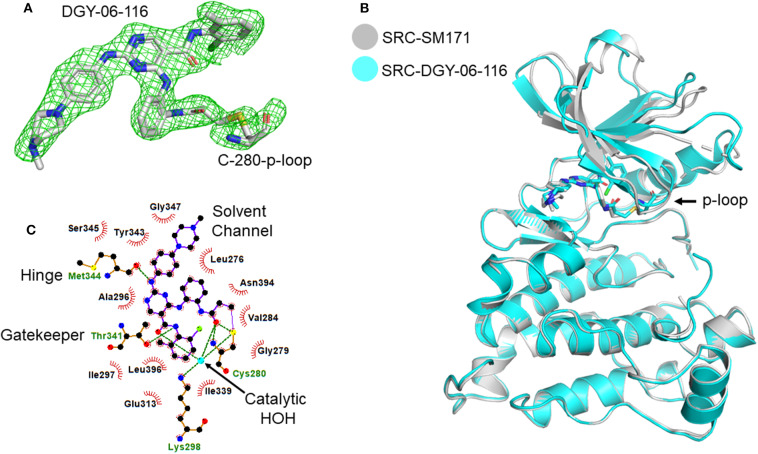
Structural characterization of the Src-DGY-06-116 complex. **(A)** A *F*_o_-*F*_c_ map at 2.5σ shows continuous electron density observed for DGY-06-116 forming a covalent linkage to Cys-280 of the p-loop. **(B)** DGY-06-116 binding to Src kinase leads to bending of p-loop Cys-280 consistent with design. **(C)** Two-dimensional representations of interactions between DGY and Src kinase domain residues. Hydrogen bonds are shown as green dashes. Hydrophobic interactions are shown as red spikes.

To further understand the nature of the interactions between DGY-06-116 and Src, we determined a co-crystal structure of DGY-06-116 with Src (PDB code: 6E6E) at 2.15 Å resolution. Data collection and refinement statistics are shown in Table 1. This structure includes eight molecules in the asymmetric unit. The main difference between individual protomers was variation in an N-terminal lobe loop conformation, but there were no differences in the conformations of the ATP binding sites (Figure S3). DGY-06-116 was easily modeled into the predicted binding site for all, with the warhead forming continuous electron density with Cys-280 (Figure 3A). As seen with SM171, the p-loop of the Src kinase is bent and thus allows a covalent bond to form, a phenomenon that is not observed with other Src structures (Figure 3B). As expected, the nitrogen of DGY-06-116′s carboxamide linker hydrogen bonds to gatekeeper Thr-338. The nitrogen is also part of a coordinated hydrogen bonding network that includes an active-site water (HOH432), catalytic Lys-298, the backbone of Cys-280, and the carbonyl DGY-06-116 (Figure 3C). The chloro-methyl phenyl substituent is situated in the back pocket of the Src kinase, creating hydrophobic interactions with Ile-297 and Leu-396. The anilinopyrimidine forms two hydrogen bonds with the backbone of Met-341 in the hinge region and the methyl piperazinyl tail extends to the solvent channel forming hydrophobic contacts with Val-284 and Gly-347 (Figure 3C). These results confirm that DGY-06-116 forms a covalent bond with Cys-280, but also suggest that hydrophobic interactions with the back pocket significantly contribute to affinity, possibly explaining the high potency of the non-covalent analog NJH-01-111.

**Table 1:** Data collection and refinement statistics.

**Crystallography statistics**	
**DATA COLLECTION**	
X-ray source	APS 19–1D
Wavelength (Å)	0.9795
Space group	P1
Unit cell	
*a, b, c* (Å)	63.53, 84.03, 120.11
α, β, γ (°)	89.96, 90.05, 90.12
Resolution (Å)	50.00–2.15 (2.19–2.15) a
Unique reflections	123,213
Redundancy	3.6 (3.0)
Completeness (%)	91.7 (91.6)
Wilson *B*-factor	32.1
*R*_merge_ (%)	12.7 (96.4)
I/σ	9.7 (1.0)
**REFINEMENT**	
Resolution	43.66–2.15 (2.19–2.15)
Reflections Used	122,649
*R*_free_ reflections	5,955
*R*_work_/*R*_free_ (%)	25.0/28.5
Non-hydrogen atoms	17,919
Protein	17,140
Water	435
Ligand	344
RMSD	
Bond lengths (Å)	0.002
Bond angles (°)	0.592
Average *B*-factor (Å^2^)	46
Protein	46.84
Ligands	31.75
Water	38.46
Ramachandran plot (%)	
Favored	95.02
Allowed	4.11
Disallowed	0.4
PDB accession code	6E6E

### Inactivation by Src by DGY-06-116 Is Slow

**Figure 4:**
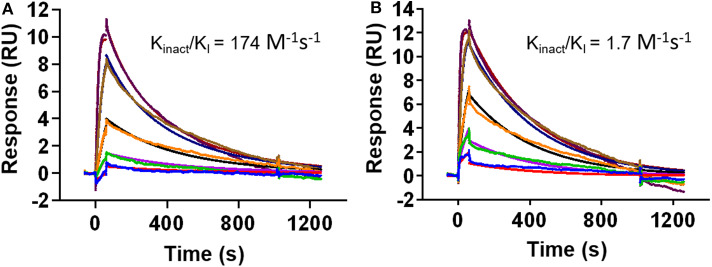
DGY-06-116 shows a slow inactivation rate. **(A)** Covalent DGY-06-116 and **(B)** non-covalent analog NJH-01-111. Data from each interaction were analyzed using both a 1:1 kinetic model and a two-state covalent interaction (p-loop movement) model for calculation of *k*_inact_/*K*_I_.

While the crystal structure clearly indicates covalent binding to Src, the enzymatic assay could not distinguish between DGY-06-116 and NJH-01-111. Nevertheless, clear differences are seen in the selectivity of these compounds (Du et al., 2020). Previously, we used the MSA to measure the inactivation rate of other covalent inhibitors (Tan et al., 2017). However, in this case, we could not because the IC_50_, 1 h of for DGY-06-116 was near the enzyme concentration used in the assay and therefore close to the theoretical sensitivity limit. To evaluate the inactivation rate, we used a surface plasmon resonance assay to estimate *k*_inact_/*K*_I_ (Copeland, 2013; Miyahisa et al., 2015). In our setup, biotinylated Src kinase was immobilized on the biosensor. Binding kinetics for DGY-06-116 and NJH-01-111 were found to be similar, indicating a similar initial binding event (Figures 4A,B). Although the decay appeared similar on visual inspection, we were unable to fit the curve for DGY using a one-state model. We considered that the p-loop must move into position to allow a covalent bond to form. We therefore used a two-state model to calculate *k*_inact_/*K*_I_. For the non-covalent inhibitor NJH-01-111, the value was only 1.7 M^−1^ s^−1^, indicating that a covalent bond did not form. In contrast, *k*_inact_/*K*_I_ for DGY-06-116 was 174 M^−1^ s^−1^, consistent with covalent bond formation. However, when comparing inactivation rate constant (*k*_inact_), DGY-06-116 showed a rate of 5.7 × 10^−7^ s^−1^, several orders of magnitude slower than for other validated covalent compounds such as neratinib (2 × 10^−3^ s^−1^) and afatinib (1 × 10^−3^ s^−1^) (Gierse et al., 1999; Papp-Wallace et al., 2014; Schwartz et al., 2014), showing modest irreversible inhibition. Given that the p-loop must shift to form a covalent bond with DGY-06-116, we speculate that this slow *k*_inact_ occurs because it depends on protein dynamics at the p-loop. This is in agreement with prior ideas about the importance of p-loop movement for p-loop targeted inhibitors (Kwarcinski et al., 2012).

## Discussion

In these studies, we established that DGY-06-116 binds covalently to Src in a manner similar to SM1-71, where the p-loop must kink to establish the covalent bond. However, we also showed that a non-covalent analog, NJH-01-111, binds with a similar high affinity. We also showed that the covalent reaction is slow. Altogether, our interpretation of these findings is that a strong reversible interaction is required to allow sufficient time for the p-loop to sample a kinked conformation compatible with covalent bond formation.

We speculate that that optimization of the interaction between the p-loop and compound may increase the inactivation rate, leading to further improvements in compound selectivity in biological systems, since covalent bond formation appears to be the major driver of selectivity (Du et al., 2020). One way to do this would be to increase the length of the linker to the covalent warhead so the p-loop does not have to kink. Computationally, this appears to be a viable strategy since simulated docking shows that extended linkers retain existing interactions and may even add additional hydrogen bonding with the main-chain oxygen of Gln-275 (Figure S4).

Despite the slow inactivation rate of DGY-06-116, this compound is a selective Src inhibitor that will enable laboratory studies of Src-driven biology, with applications in cancer. One potential application may relate to the subject of acquired resistance, which is a major challenge with kinase inhibitors (Lovly and Shaw, 2014). Src has been implicated in mechanisms that underlie the development of hormone therapy resistance in breast cancer (McDonnell and Norris, 2002; Hiscox et al., 2006) and resistance to chemotherapy in triple-negative breast cancer (Wu et al., 2016). Src is also involved in drug resistance to Her2-directed therapy and for certain head and neck and lung cancers (Carretero et al., 2010; Sen et al., 2011). Src has also been implicated in non-small cell lung cancers harboring mutations in EGFR where Src is activated via Cripto-1 (Park et al., 2014). Indeed, this concept is being tested in a clinical trial (NCT02954523) where dasatinib and osimertinib are delivered together. Another possible application is in KRAS-mutated lung cancer, where loss of the Lkb1 tumor suppressor activates Src signaling. In mouse cancer models that mimic this cancer state, the combined inhibition of Src, PI3K, and Mek showed synergistic tumor regression (Carretero et al., 2010). Finally, a lack of predictive biomarkers has limited prior Src-directed trials (Puls et al., 2011). Our tool compound may allow us to identify cancer populations that are sensitive to Src inhibition through chemistry-first biomarker discovery approaches (McMillan et al., 2018).

## Accession Codes

The atomic coordinates and structure factors have been deposited in the RCSB Protein Data Bank archive (PDB) for human proto-oncogene tyrosine-protein kinase Src in complex with DGY-06-116 (PDB ID: 6E6E). *SRC*_HUMAN P12931.

## Data Availability Statement

This article contains previously unpublished data. The name of the repository and accession number(s) are not available.

## Author Contributions

NG, TZ, KW, and DG: Conceptualization. GD and NH: Chemistry. DG: Structure determination and biochemistry. TZ, DG, KW, and NG: Writing. The manuscript was edited through contributions of all authors. All authors have given approval to the final version of the manuscript.

## Conflict of Interest

KW is on the SAB for Vibliome Therapeutics. NG is a founder, SAB, and equity holder in Gatekeeper, Syros, Petra, C4, B2S, and Soltego. The Gray lab receives/has received funding from Novartis, Takeda, Astellas, Taiho, Janssen, Kinogen, Voronoi, Her2llc, Deerfield, and Sanofi. GD, NH, SR, DG, TZ, KW, and NG are inventors on a Src covalent inhibitor patent. Information regarding Src is accessible via UniProtKB P12931. The remaining author declares that the research was conducted in the absence of any commercial or financial relationships that could be construed as a potential conflict of interest.

## Figures and Tables

**Table:** 

**Reagent or resource**	**Source**	**Identifier**
**BACTERIAL STRAINS**		
*E. coli* BL21DE3 cells	Gift from Dr. M. Seeliger (SUNY, Stony Brook)	N/A
Src kinase purification, assays, and crystal structure		
pET28a Src kinase expression vector	Gift from Dr. M. Seeliger (SUNY, Stony Brook)	N/A
Custom oligos:AAGCTGGGGCAGGGCAGCTTTGGAGAGGTCTGG	Sigma-Aldrich	N/A
QuikChange II Site-Directed Mutagenesis Kit	Agilent Technologies	Cat # 200524
Kanamycin	Sigma-Aldrich	Cat # K1377
Streptomycin	Sigma-Aldrich	Cat # S9137
Chloramphenicol	Sigma-Aldrich	Cat # C0378
Bosutinib	Sigma-Aldrich	Cat # PZ0192
SM1-71	Prof. Nathanael Gray, DFCI, Harvard	https://graylab.dana-farber.org/
DGY-06-116	Prof. Nathanael Gray, DFCI, Harvard	https://graylab.dana-farber.org/
NJH-01-111	Prof. Nathanael Gray, DFCI, Harvard	https://graylab.dana-farber.org/
LabChip® EZ Reader automated microcapillary electrophoresis platform	PerkinElmer	https://www.perkinelmer.com/product/assy-ez-reader-ii-ship-level-122919
Fluorescently labeled peptide-4 substrate	Perkin Elmer	https://www.perkinelmer.com/product/dkp-peptide-4-packed-760348
EZ-Link™ Sulfo-NHS-Biotinylation Kit	ThermoFisher	Cat # 21425
Biacore S200	GE Healthcare	https://www.gelifesciences.com/en/us/shop/protein-analysis/spr-label-free-analysis/systems/biacore-s200-p-05541
Biotin Capture Kit and Sensor Chip CAP	GE Healthcare	Cat # 28920233
Deposited data		
Src-DGY-06-116	https://www.rcsb.org (this study)	PDB ID: 6E6E
**SOFTWARE AND ALGORITHMS**		
EZReader 3.0 software	Perkin Elmer	https://www.perkinelmer.com/lab-products-and-services/resources/software-downloads.html
GraphPad software version 7	GraphPad Software	www.graphpad.com
HKL3000	(Minor et al., 2006)	https://sbgrid.org/
PHENIX 1.14rc3_319	(Adams et al., 2010)	https://sbgrid.org/
Coot	Emsley and Cowtan, 2004	https://sbgrid.org/
CCP4 7.0	Winn et al., 2011	https://sbgrid.org/
Pymol 2.3	Schrodinger, LLC	https://sbgrid.org/
LIGPLOT	Laskowski R A, Swindells M B (2011)	https://www.ebi.ac.uk/thornton-srv/software/LigPlus/
Biacore™ insight evaluation software	GE Healthcare Life Sciences, USA.	https://www.gelifesciences.com/en/us/shop/protein-analysis/spr-label-free-analysis/systems/biacore-s200-p-05541

## Supplementary Material

Figure S1 Src kinase domain architecture and location of targetable cysteine the p-loop.

Figure S2 Src^C280S^ efficiently phosphorylates the peptide substrate. Percent conversion from non-phosphorylated to phosphorylated substrate is shown over time for a range of Src concentrations. Each data point represents a triplicate measurement. **(A)** WT Src. **(B)** Src^C280S^.

Figure S3 Differences between protomers in the asymmetric crystallographic unit. All 8 protomers were superimposed and each colored differently.

Figure S4 Covalent docking model predicts hydrogen bonding interactions for linker optimization.

## Method Details

### Expression, Purification and Crystallization of Human Src Kinase and Cys_280_-Ser Mutant Src Kinase

The Src (UniProtKB P12931) kinase domain was purified as reported previously (Rao et al., 2019). The Cys280-Ser mutant Src version was generated via site-directed mutagenesis and purified similarly.

### Enzymatic Assay for IC_50_ Determination

An automated microcapillary electrophoresis platform (PerkinElmer LabChip® EZ Reader) capable of separating and detecting fluorescently labeled peptides based on charge (Elkins et al., 2016) was used for measuring Src kinase activity. Briefly, purified Src enzyme, fluorescently labeled peptide-4 substrate (purchased from Perkin Elmer), ATP, and inhibitors (SM1-71, Bosutinib, DGY-06-116, and NJH-01-111) were combined into a single well, incubated for 1 h incubation following phosphorylation of the substrate. Substrate (non-phosphorylated peptide) to product (phosphorylated peptide as a result of Src kinase activity) conversion was measured over time. The kinase reaction buffer consisted of 100 mM Hepes, pH 7.3, 0.015% Brij-35, 0.004% Tween-20, and 10 mM MgCl_2_. Peptides were separated using running buffer made of 100 mM Hepes, pH 7.3, 0.015% Brij-35, 1 mM disodium EDTA, 0.1% coating reagent 3, 5% DMSO, and 1× coating reagent 8. Final reaction conditions included 1.25 nM of purified Src kinase, 100 μM ATP, and 1 μM peptide-4 substrate. The percent conversion was determined by EZReader 3.0 software and analyzed using GraphPad software version 7 (La Jolla, California, USA) for determination of IC_50_ values.

### Crystallization and Structure Determination of Src-DGY-06-116 Complex

Purified Src kinase was concentrated to 10 mg/ml and incubated with three fold excess of inhibitor DGY-06-116 at room temperature for 1 h. Co-crystals of Src-DGY-06-116 complex were obtained by vapor diffusion from a hanging drop setup at 20°C using 0.1 M magnesium formate dihydrate and 15% PEG3350 as precipitant. Crystals appeared overnight and were harvested after 2 days in mother liquor with 30% glycerol before freezing in liquid nitrogen. Diffraction data were collected using beamline 19-ID of the Advanced Photon Source, Argonne National Laboratory and scaled using HKL3000 (Minor et al., 2006). Molecular replacement solution was obtained using Phaser (McCoy et al., 2007) and 4MXO as initial search model. Model building and refinement were carried out using Coot (Emsley and Cowtan, 2004) and Phenix (Adams et al., 2010).

### SPR Based Kinetics of Covalent Inhibition

A Biacore S200 was used to evaluate binding of covalent inhibitor DGY-06-116 and its non-covalent analog NJH-01-111 with Src kinase. Src was biotinylated using EZ-Link™ Sulfo-NHS-Biotinylation Kit (ThermoFisher). Biotinylated Src kinase was captured using the Biotin Capture Kit and Sensor Chip CAP (GE Healthcare). The Biotin Capture Kit works with a single-stranded DNA sequence on Sensor Chip CAP. A capture reagent consisting of a biotinylated complementary DNA strand and streptavidin is captured by the single-stranded DNA, allowing capture of other biotinylated molecules by streptavidin. At the end of the binding cycle, the DNA duplex is chemically disrupted, washing away all materials except the original single-stranded DNA sequence. The sequence is repeated for each binding cycle of the assay. Src was captured at surface densities between 800 and 900 RU for each cycle of the assay. Dilutions of DGY-06-116 and NJH-01-111 from 100 to 1.2 nM were made in running buffer (100 mM HEPES pH 7.3, 0.015% Brij-35, 0.004% Tween-20, 10 mM MgCl_2_, and 1% DMSO) and tested for binding using a multi-cycle assay. The SPR data were analyzed using Biacore™ Insight Evaluation Software (GE Healthcare Life Sciences, USA). Data from each interaction were analyzed using both a 1:1 kinetic model and a two-state covalent interaction model (involving p-loop movement) for calculation of *k*_inact_/*K*_I_ using Biacore S200 Evaluation software (GE Healthcare Life Sciences, USA).
