# Supplementary figures and images for: Structure and Characterization of a Covalent Inhibitor of Src Kinase
Source: Front Mol Biosci. 2020 May 19;7:81. doi: 10.3389/fmolb.2020.00081 (PMC7248381; doi:10.3389/fmolb.2020.00081)

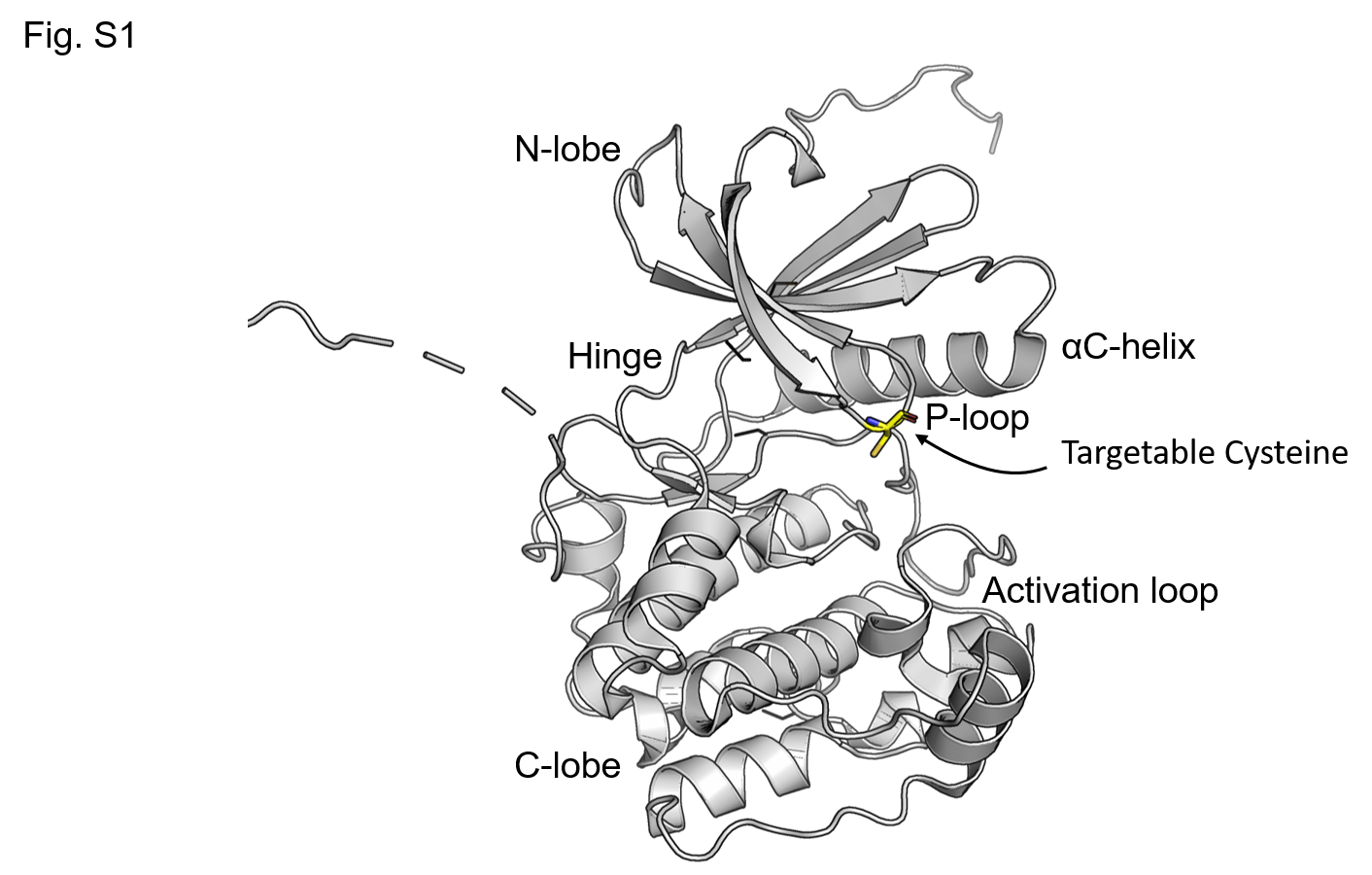

Supplement: Figure S1 — Src kinase domain architecture and location of targetable cysteine the p-loop. [file Image_1.TIF]

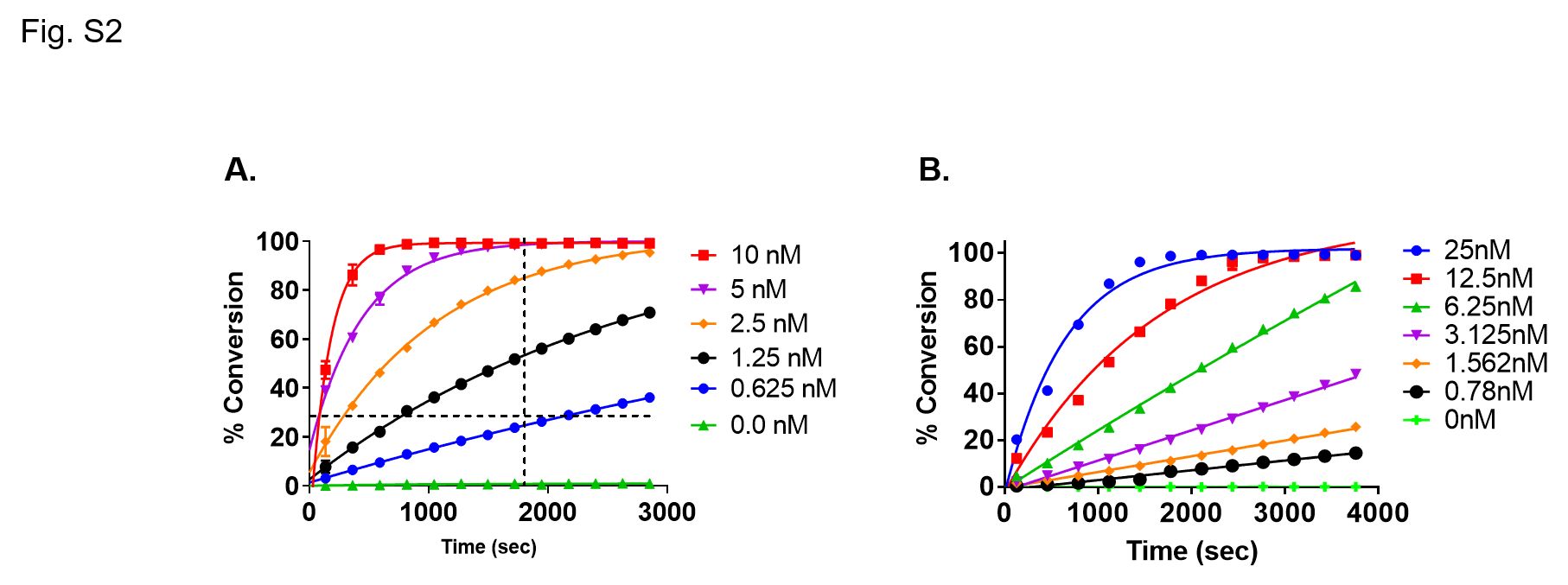

Supplement: Figure S2 — SrcC280S efficiently phosphorylates the peptide substrate. Percent conversion from non-phosphorylated to phosphorylated substrate is shown over time for a range of Src concentrations. Each data point represents a triplicate measurement. (A) WT Src. (B) SrcC280S. [file Image_2.TIF]

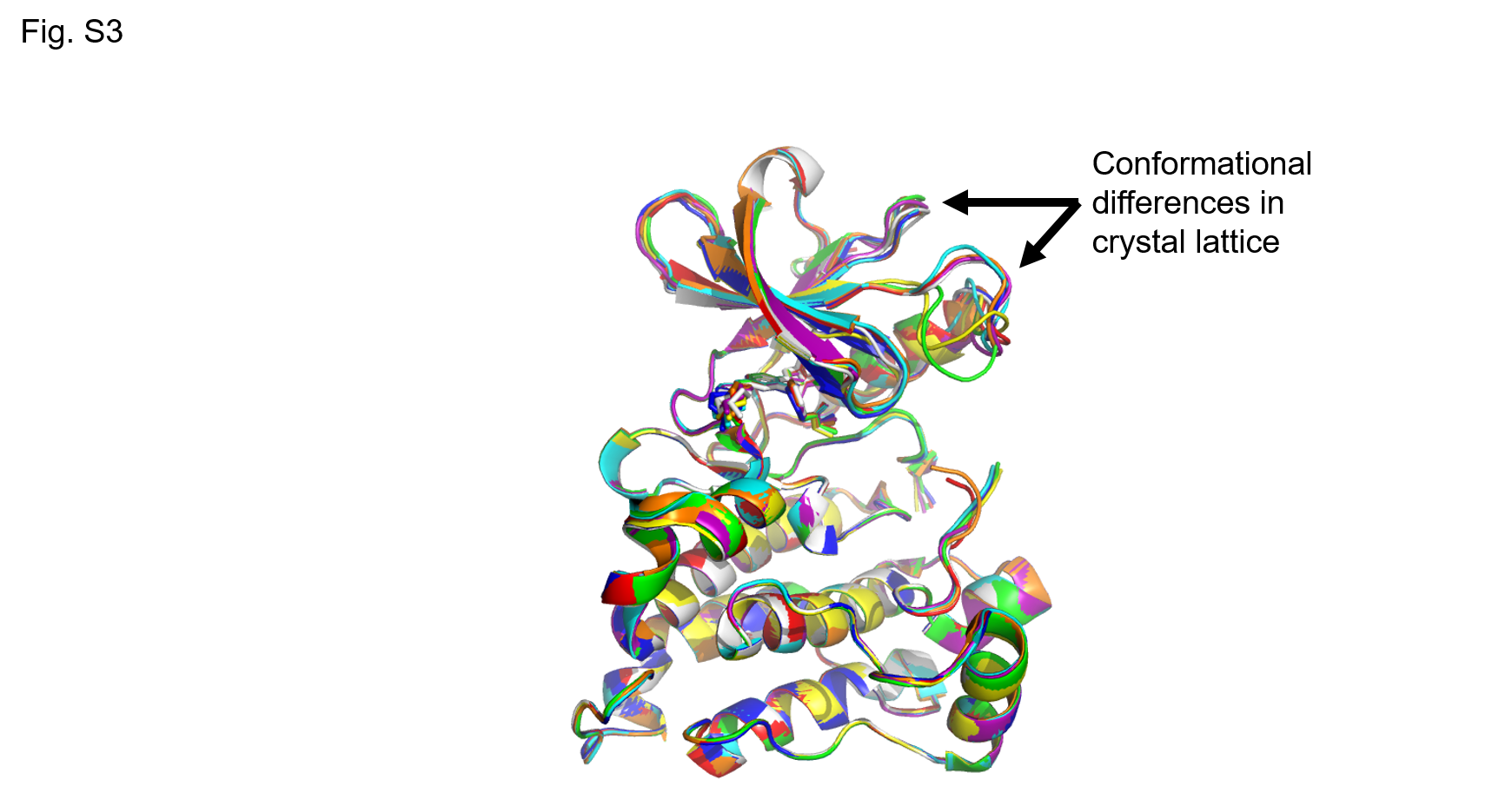

Supplement: Figure S3 — Differences between protomers in the asymmetric crystallographic unit. All 8 protomers were superimposed and each colored differently. [file Image_3.TIF]

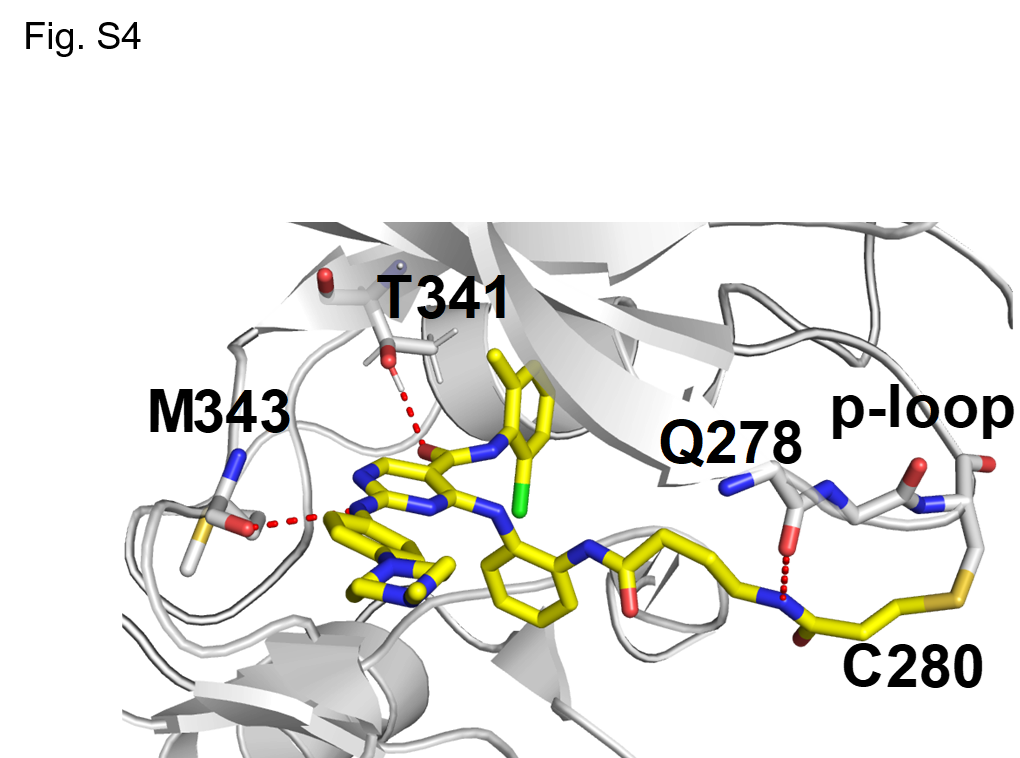

Supplement: Figure S4 — Covalent docking model predicts hydrogen bonding interactions for linker optimization. [file Image_4.TIF]
